# Scaling the fitness effects of mutations with respect to differentially adapted *Arabidopsis thaliana* accessions under natural conditions

**DOI:** 10.1093/evolut/qpaf029

**Published:** 2025-02-16

**Authors:** Frank W Stearns, Juannan Zhou, Charles B Fenster

**Affiliations:** Department of Biology, Stevenson University, Owings Mills, MD, United States; Department of Biology, University of Florida, Gainesville, FL, United States; Department of Biology and Microbiology, South Dakota State University, Brookings, SD, United States; Oak Lake Field Station, South Dakota State University, Astoria, SD, United States

**Keywords:** *Arabidopsis*, mutations, adaptation genetics, EMS, Bayesian hierarchical model, nonparametric model

## Abstract

Mutations are the ultimate source of genetic variation for natural selection to act upon. A major question in evolutionary biology is the extent to which new mutations can generate genetic variation under natural conditions to permit adaptive evolution over ecological time scales. Here, we collected fitness data for chemically induced (ethylmethane sulfonate, EMS) mutant lines descended from two *Arabidopsis thaliana* ecotypes that show differential adaptation to the local environment of our common garden plot. Using a novel nonparametric Bayesian statistical approach, we found that both ecotypes accumulated substantial proportions of beneficial mutations. The poorly adapted ecotype exhibited higher mean mutational effects and higher variance in the fitness effects of mutations compared to the well-adapted ecotype. Furthermore, we predict that it takes less than 4,000 generations for the fitness space of the two ecotypes to overlap through mutation accumulation, and that a single founder, through mutation accumulation, is able to achieve the species-wide genetic variation in less than 10,000 generations. Our results provide evidence for relatively rapid local adaptation of *Arabidopsis thaliana* in natural conditions through new mutations, as well as the utility of a nonparametric Bayesian method for modeling the distribution of fitness effects for field-collected data.

## Introduction

Since mutations are the ultimate source of genetic variation, quantifying mutation parameters is fundamental to understanding evolutionary processes such as adaptation genetics (Fisher, 1930). A key mutation parameter is the distribution of mutation effects on fitness (DFE) (Eyre-Walker & Keightley, 2007; Halligan & Keightley, 2009). Because the vast majority of new mutation studies have been conducted on microorganisms under laboratory conditions (Eyre-Walker & Keightley, 2007) we lack estimates of this parameter in macro-organisms under natural conditions where the effects of mutations can be assessed in the environment where natural selection occurs. Whether mutations have beneficial effects on fitness traits rarely or more frequently and the size of these effects scaled to environmental effects on the trait(s) will determine the role of new mutations in a population’s response to selection. This has implications for use in crop production (e.g., Chaudhary et al., 2019; Gaines et al., 2020) and whether populations can evolve in place in response to the stresses imposed by anthropogenic alteration of the environment, including climate change (Frankham et al., 2017).

One approach to studying mutations is spontaneous mutation accumulation. Mutation accumulation lines (MA lines) have been used since at least 1928 (Muller) to study the cumulative effects of mutations on traits, including fitness (Crombie et al., 2024; Halligan & Keightley, 2009; Katju & Bregthorsson, 2019). MA lines are lines that are derived from a common nearly homozygous founder, that are cultivated via intense inbreeding (full sib mating if separate sexes, or selfing, if hermaphrodites) or by using clonally derived haploid organisms and thus, genetically diverge through the accumulation of independent mutations (Lynch & Walsh, 1998). Using the MA line approach in *A. thaliana*, lab-based estimates of the contribution of mutations to heritable genetic variance (*h*^2^_*m*_, Lynch & Walsh, 1998; Shaw et al., 2000) are typically 10-fold higher than field-based estimates (Roles et al., 2016; Rutter et al., 2010, 2018), with 500 and 5,000 generations based on lab and field-based estimates, respectively, needed for mutation to generate a typical *h*^2^ of 50%. This reflects the much greater environmental variance in field versus lab settings.

While field-based estimates of *h*^2^_*m*_ are low, a number of studies indicate that mutations have the potential to contribute to population genetic variation in a meaningful way. First, field-based studies with *Arabidopsis thaliana* reveal that a significant proportion of new mutations are beneficial (Roles et al., 2016; Rutter et al., 2010, 2018; Weng et al., 2021) although how specific mutations affect fitness is dependent on the environmental context (Rutter et al., 2018; Stearns & Fenster, 2016a,b; Weng et al., 2021). Second, the amount of genetic variation generated by mutations can, in a relevant number of generations, generate levels of genetic variation in *A. thaliana* seen within and between populations (Rutter et al., 2018; Weng et al., 2021). Third, there is a direct link between the likelihood of a site mutating and whether that site is polymorphic in natural populations of *A. thaliana* (Monroe et al., 2022; Weng et al., 2019), suggesting that such studies are biologically meaningful.

The above evidence suggests that mutations have the potential to contribute to the evolution of adaptations through their response to selection at microevolutionary time scales. However, the use of MA lines to represent new mutations and the subsequent analyses limit inference of mutational input to adaptive response. Even using the model system *A. thaliana*, with generation times of 3–4 per year, it is difficult to produce many generations of mutation accumulation. All field studies to date to assess the fitness effects of mutations in *A. thaliana* have been limited to 8-25 generations of mutation accumulations. Second, previous studies of mutation accumulation, whether in the field (above citations) or lab (Rutter et al., 2010; Shaw et al., 2002), infer the mutation effect on fitness by comparing the distribution of line fitness relative to the founder. Parametrizing mutation effects have required the DFE to be within a designated family of probability distributions (e.g., Gamma distribution) and impose prior distributions (e.g., Eyre-Walker & Keightley, 2009; Rutter et al., 2010; Shaw et al., 2002). This practice relies on strong assumptions of the shape of the DFE, and therefore could potentially introduce bias. Finally, the contribution of mutation to population differentiation scaled to actual population genetic differentiation has been inferred (Rutter et al., 2018) and only rarely directly included in experimental designs (Weng et al., 2021). Consequently, we have limited understanding of the potential contribution of individual mutations to the patterns of genetic differentiation commonly observed in nature.

Here, we use a novel nonparametric Bayesian hierarchical modeling approach to estimate the DFE and magnitude of mutation effects under field conditions for two ecotypes of *Arabidopsis thaliana* using chemically induced (ethylmethane sulfonate, EMS) mutation lines and four reference ecotypes with no associated mutant lines. Our approach allows us to avoid potential bias to determine the effect of starting fitness on frequency and size of beneficial mutations. We also calibrate the effect of mutations in fitness space by testing whether mutations alone can change the performance of one accession to be equivalent to another of higher performance. Finally, our use of EMS lines allows us to quantify the total effects of many more mutations relative to MA line approaches relying on new mutational events. Sampling many simultaneous mutations may allow us to better estimate the distribution of effects of mutations because we are able to make assumptions in our modeling approach by exploiting the Central Limit Theorem. Consequently, this approach enables us to estimate the mean and standard deviation of the DFE without making a priori assumptions about the shape of the DFE. Our Bayesian model also relies on several other innovative approaches, including accounting for covariance structures between sublines due to segregating mutations, as well as the usage of a logistic function to model survival probability as a function of genetic values, allowing us to simultaneously fit all fitness data that contains many zeros due to nonsurviving seedlings.

## Methods

### *Arabidopsis thaliana* accessions

We used six different accessions of *Arabidopsis thaliana* for our study. Two accessions were chosen as focal accessions to undergo mutagenesis. These accessions were chosen to represent high and low fitness in our experimental habitat based on previous work (Stearns & Fenster, 2016a). These accessions were COL (Missouri, USA) and CS76116 CVI (Republic of Cabo Verde). Four other accessions were chosen based on current data on relatedness from the Borevitz lab (http://www.naturalvariation.org/hapmap). Among these four accessions, two were closely related to COL, and two were closely related to CVI. This allowed us to examine the genetic space around each focal accession. These were CS28051 (Arby, Sweden), CS28364 (Jena, Germany), CS28510 (Solomennoye, Russia), and CS76197 (Niederzenz, Germany). Founder sublines were developed without mutagenesis for each of these four.

### Mutagenesis

Mutagenesis followed the methods from Stearns and Fenster (2016b). Briefly, seeds were collected from a single individual for each accession. Seeds were then treated with a 20 μM solution of ethylmethane sulfonate (EMS), an alkylating agent that induces G:C to A:T substitutions (Greene et al., 2003). The spectrum of mutations produced by EMS is similar to that of spontaneous mutations, although it does not include indels (Greene et al., 2003). The spontaneous mutation rate for indels is 1.3 × 10^−9^ compared to 7 × 10^−9^ for SNMs for the Columbia line (Weng et al., 2019); thus EMS mutagenesis accounts for about 84% of the normal mutation spectrum (7 × 10^−9^/(1.3 × 10^-9^ + 7 × 10^−9^)). Based on the proportion of siliques segregating albino seed (36%), we estimated 25 mutations per cell in coding regions per genome (Stearns & Fenster, 2016b), which is very similar to the estimate made by (Camara et al., 2000). The basis of this estimate was originally derived by Korneef et al. (1982). We are not aware, at this time, of any direct sequencing attempts to directly relate EMS dosage with sequence mutation rate. The mutation rate we calculate based on the indirect method resulted in an approximate six-fold greater number of nonsynonymous mutations in protein-coding regions (Weng et al., 2019) as quantified from direct sequencing of Columbia mutation accumulation lines representing 25 generations of spontaneous MA (Ossowski et al., 2010). We therefore expect our lines to carry the number of mutations roughly equivalent to 150 generations of mutation accumulation.

To determine if EMS-induced mutation rate is different between the founders. We applied 20 µM EMS to 20 and 14 siliques from Cape Verde (CV) and Columbia, respectively. We observed 5 out of 14 siliques having the albino phenotype for Columbia and 3 out of 20 siliques with albinos for CV. Fisher’s exact test showed no significant difference between the albino frequency between the two founders.

**Figure 1.**
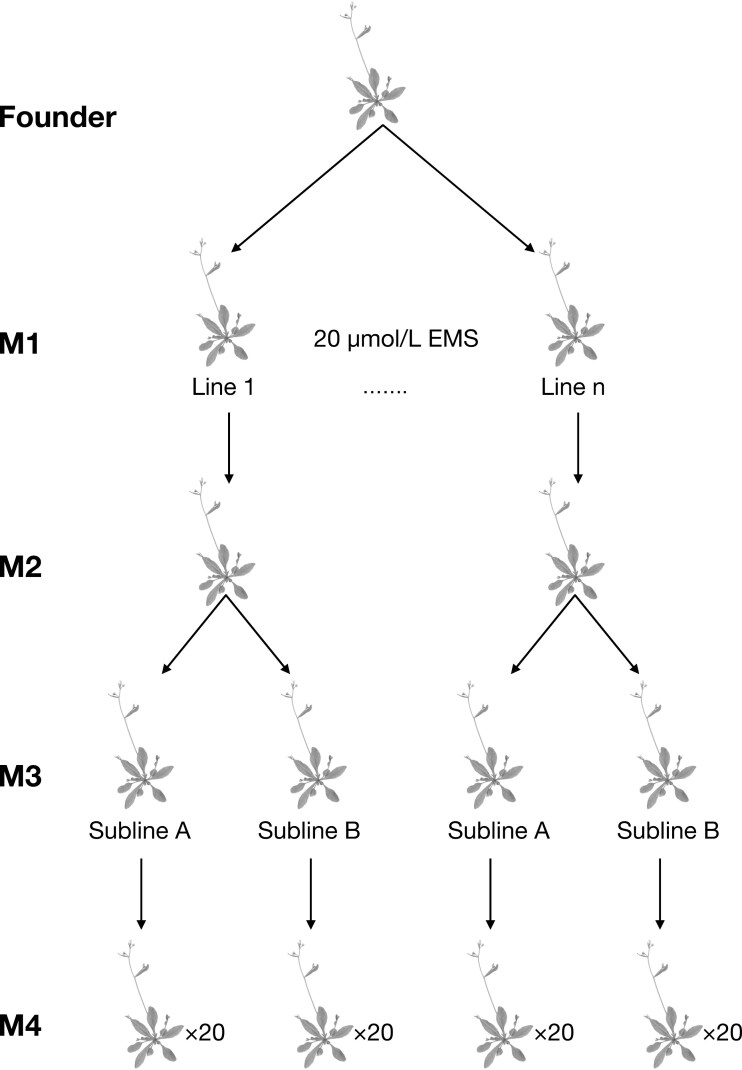
A schematic representation showing the process of generating mutation sublines from *Arabidopsis thaliana*. Seeds were collected from a single Columbia founder and treated with 20 μmol/L ethylmethane sulfonate (EMS) for 12 hr. Twenty and 16 mutant lines were derived from this treatment for the Columbia and Cape Verde founders, respectively. Each line was then split into two sublines each (M3 generation), and seeds from these sublines (via selfing) were planted in the field (Beltsville Experimental Agricultural Station (UMD) in Beltsville, MD) (N 39.05378 W −76.95387).

Mutant and founder lines were established from single seeds with/without EMS treatment. The lines were raised in growth chamber conditions (Stearns & Fenster, 2016b) and allowed to self-fertilize for two generations (generation M1 and M2, Figure 1). They were then split into two sublines (generation M3) to account for maternal effects and allowed to self-fertilize for an additional generation to produce the seeds for plants used in this experiment (M4 generation). This breeding design results in 87.5% homozygosity, so we expect little residual heterogzygosity based from either EMS treatment or from the original premutation founders (Monroe et al., 2022; Weng et al., 2019).

### Fitness assessment

All seedlings were planted at the Beltsville Agricultural Station (UMD) in Beltsville, MD on one day during the Fall of 2014. The timing of planting corresponds to the predominant winter annual life cycle that is exhibited by *A. thaliana* throughout its range (fall germination, overwinter as a vegetative rosette, spring flowering, and senescence). Average low temperatures are several degrees below freezing for January and February at this site. This field was surrounded by 10-foot (3-m) fencing to preclude deer foraging. Preparation and planting followed Stearns and Fenster (2016a), which includes removing preexisting vegetation and raking the soil. Seedlings were planted 10 cm apart at the 2- to 4-leaf stage. There were 60 plants for each subline of both mutant lines and premutation founders (with few exceptions, Table 1) for a total of 5,520 plants. For all founders, we planted 120 seedlings for the control. For Columbia, we planted an additional 2,400 mutated seedlings (20 mutant lines). For CV, we planted 1,920 mutated seedlings (16 mutant lines).

**Table 1.** Summary statistics of mutation experiments for the two founders, Columbia (COL) and Cape Verde (CV) subjected to both the mutation and control treatment and the four founders (28051, 28364, 28510, 76197) subjected to only the control treatment. Log_10_ mg dry weight was calculated only for the survived seedlings.

Population	No. lines		No. seedlings		Survival rate		Log_10_ dry weight in mg (mean ± 1sd)	
	Control	EMS	Control	EMS	Control	EMS	Control	EMS
COL	1	20	120	2,400	0.51	0.55	2.892 ± 0.451	2.909 ± 0.377
CV	1	16	120	1,920	0.35	0.33	2.143 ± 0.531	2.222 ± 0.459
28051	1	0	120	0	0.14	–	2.912 ± 0.457	–
28364	1	0	120	0	0.58	–	3.115 ± 0.305	–
28510	1	0	120	0	0.49	–	3.140 ± 0.380	–
76197	1	0	120	0	0.45	–	2.971 ± 0.384	–

*Note*. EMS, = ethyl methanesulfonate; COL = Columbia; CV = Cape Verde.

The plots were initially watered for a week to help with establishment but otherwise exposed to natural conditions. Plants were harvested in May of 2015 when all plants had senesced. Plants were then dried in heat chambers. Total seed set for an annual plant is a standard method of quantifying fitness in plant ecological genetics (Briggs & Walters, 2016; Schemske, 1984). Measuring seed production is a robust estimate of reproductive success since *A. thaliana* is highly selfing (e.g., Stenøien et al., 2005). Above-ground biomass has been shown to be strongly correlated to fruit number in earlier experiments with these lines (Shaw et al., 2000; Stearns & Fenster, 2016b,a; Weng et al., 2021). Stearns and Fenster (2016b) found a correlation of *r*^2^ = 0.89 (*n* = 15 plants). Therefore, biomass was used as an estimate of reproductive output. Thus, we were able to efficiently quantify the DFE of new mutations measured in an environment approximating natural selection. Many previous experiments to study the fitness effects of new mutations using microorganisms are constrained to laboratory environments (e.g., Baer et al., 2006), but do have the advantage of measuring fitness as population growth rates, which is an arguably more direct estimate of fitness.

### Bayesian inference of genetic and nongenetic parameters

Our main interest in this paper is to estimate the parameters for the distribution of fitness effects (DFE) of mutations from the fitness measurements.

Here, we propose a novel nonparametric Bayesian approach for characterizing the DFE for the two studied ecotypes. This framework allows us to solve several key challenges in modeling our fitness data. Specifically, it allows us to exploit the Central Limit Theorem to estimate the mean and standard deviations of the DFE without having to make a priori assumptions about its shape, thereby alleviating a potential source of bias. It also allows us to easily model the complex correlation in the fitness effects of mutations across individuals due to segregating mutations in the intermediate generations, M2 and M3 (see Figure 1). Additionally, it naturally models the zero-inflated fitness data due to high mortality rates.

We begin by presenting the mathematical model that describes the relationship between fitness measurements and genetic as well as nongenetic factors. We then introduce the practical inference procedure. Detailed mathematical derivations of the model and the Bayesian inference process are provided in the Supplementary Material.

We decompose the fitness measurements of M4 individuals (Figure 1) as a sum of genetic and nongenetic terms:


$$\begin{array}{l} z_{ijkl}\;=y_{i}+m_{ijk}+h_{ijkl}\;+\;b_{l}+\varepsilon_{ijkl}.\; \end{array}$$ (1)

Here $z_{ijkl}$ is the measurement of founder i, line j, subline k, and individual l. Specifically, $z_{ijkl}\;$ equals zero if the individual did not survive to the point of measurement, otherwise is equal to the log_10_ transformed dry weight. The log transformation of dry plant weight ensures the normality of the distribution of fitness measurements and easier convergence of our inference models. Furthermore, since this approximation $10^{a\;+\;x\;}\;\approx 10^{a\;}+\;c\;\times \;x$ (where $c\;=\;log10\;\times 10^{a}$) holds when x  is small, assuming mutations mostly have relatively small effects, we can reasonably assume that the action of simultaneous mutations act additively on both the log_10_ and the original scale.

$y_{i}\;$ is the genetic value of the founder i, equal to its additive genetic component of fitness that can be passed on to its progenies. $m_{ijk}$ is the subline specific maternal effect. $h_{ijkl}$ is the deviation in the offspring’s genetic value from the founder caused by novel EMS-induced mutations. $b_{l}$ is the block effect associated with individual l. $\varepsilon_{ijkl}$ is the residual due to phenotypic and measurement noise.

The deviation in genetic value $h_{ijkl}$ of different individuals is of primary interest as it results from novel mutations introduced by EMS and is the cause of the genetic variation across individual within the same line.

To model $h_{ijkl}$, we assume that there is no epistasis and dominance. Additivity of mutations on fitness in *A. thaliana* has been documented for later life-history traits (Shaw & Chang, 2006). We also expect that 87.5% of the mutations will be homozygous because of the three generations of selfing to create the mutant lines, so that dominance will likely account for a small proportion of the observed genetic variation even when present. Additivity of new mutations of small effect has been commonly found in previous studies (Lynch, 1988). Furthermore, additive models often provide reasonably good fit to the data even in the presence of epistasis (Zhou & McCandlish, 2020; Zhou et al., 2022). We also have no reason to believe that there is residual variance in the premutation founders (Monroe et al., 2022; Weng et al., 2019).

As a result, $h_{ijkl}$ can be decomposed as a sum of effects of mutations randomly drawn from the same DFE shared by all lines derived from the same founder. We assume that the number of mutations contributing to $h_{ijkl}$ follows a Poisson distribution (Shaw et al., 2002) with the mean given by the mutation rate calculated in the previous section (25 mutations in coding regions). The distribution of SNMs per MA line fits both Poisson and negative binomial distributions, with no evidence of overdispersion from natural occurring mutations (Weng et al., 2019); thus using the same assumption for EMS-derived MA lines seems appropriate. Based on this and the additivity assumption, $h_{ijkl}$ can be expressed as a compound Poisson distribution


$$\begin{array}{l} h_{ijkl}=\underset{m=1}{\overset{P}{\sum }}\;n_{m}\times e_{m}. \end{array}$$ (2)

Here P is the total number of mutations, which is a Poisson-distributed random variable with mean λ=25. $n_{m}$ is the dosage of the mutant allele for mutation *m*, which is 0 if the mutation is lost, 1 if the mutation is still segregating, and 2 if the mutation becomes fixed. $e_{m}$ is its fitness effect, which is drawn from founder-specific DFE.

Given the moderately large number of contributing mutations, we can accordingly use the Central Limit Theorem to show that the distribution of $h_{ijkl}$ can be well approximated by a normal distribution, regardless of the shape of the DFE (see Supplementary Figure 1 for results of numerical simulations). This allows us to take a nonparametric approach to estimate the mean μ and standard deviation σ of the DFE without restricting to certain distribution families. Specifically, we can show that $h_{ijkl}$ can be approximated by a normal distribution with mean $\lambda \times \mu_{\;i}$, and variance $1.875\times \lambda \times \left({\mu^{2}}_{i}\;+\;{\sigma^{2}}_{i}\right)$, where $\mu_{\;i\;}$ and $\sigma_{\;i}$ are the mean and variance of the effects of mutations for founder $i_{\;}$. This thus provides the key result for us to characterize the founder-specific DFEs without having to specify the shape of the DFE, thereby reducing a potential source of bias.

However, simple implementation of this result is not suitable for our data as fitness between individuals in the same line and sublines additionally exhibit correlation due to shared mutations. Therefore, in practice, we further decompose $h_{ijkl}$ into three normally distributed terms in order to account for the covariance among individual fitness values, one for mutations fixed in M2 that are shared between the two sublines, one term for mutations shared among individuals within a subline, and an individual-specific residual term (see Supplementary Material for detailed derivation). And all three terms are functions of $\mu_{\;i\;}$ and $\sigma_{\;i}$, allowing us to estimate these two parameters indirectly from the fitness data.

One challenge in implementing this sampling strategy is that our fitness measurements contain many zeros since many seedlings or juvenile plants died before measurements could be made. To solve this problem, we re-formulate our observation $z_{ijkl}$ to follow a mixture distribution. We do this by modeling the probability of surviving to the time of measurement for every plant ijkl as a logistic function of the sum of its genetic effects and maternal effects. That is


$$\begin{array}{l} p_{ijkl}\;=\frac{1}{1\;+\;e^{c\left(y_{i}+m_{ijk}+h_{ijkl}\;+\;b_{l}\right)\;+\;d}}. \end{array}$$

Here c  and d are parameters that determine the shape of the logistic function that are inferred from the data. With this modification, $z_{ijkl}$ is equal to zero with probability $1\;-\;p_{ijkl}$. In the event that the seedling survived to measurement with probability $p_{ijkl}$, $z_{ijkl}$ is nonzero and follows a normal distribution with mean equal to $y_{i}+m_{ijk}+h_{ijkl}\;+\;b_{l}$, and variance equal to the variance of the noise term $\varepsilon_{ijkl}$.

In order to estimate the DFE, as well as the distributions of maternal effects and residual noise, we use a hierarchical Bayesian approach to model the random effects ($m_{ijk}$, $h_{ijkl}$, $b_{l}$, $\varepsilon_{ijkl}$) in Equation 1. In particular, we specify that a random genetic or nongenetic effect is drawn from a probability distribution (e.g., the DFE for effects of individual mutations), whose parameters (e.g., the mean and standard deviation of the DFE) in turn follow prior distributions specified by their respective hyperparameters, which are typically chosen to be uninformative. Note that in principle, we can also fit Equation 1 using linear mixed models while accounting for the zero-inflated fitness data (e.g., hurdle models, Min & Agresti, 2005). Here, we chose to use the hierarchical Bayesian model due to its high flexibility, more intuitive model specification, and ability to infer the full posterior distributions.

Additionally, we specify the random maternal effect $m_{ijk}$, block effect $b_{l}$, and the noise term $\varepsilon_{ijkl}$ to follow zero-mean normal distributions, whose standard deviations are drawn from uninformative priors. The founder’s genetic value $y_{i}$ is treated as a fixed effect and sampled from an uninformative zero-mean normal distribution with fixed standard deviation.

Ideally, we would like to perform Monte Carlo sampling for both the model parameters and individual random effects. However, this is not practical due to the complexity of our model. Therefore, to estimate the posterior distribution for the model parameters, we use the variational Bayesian method (Fox & Roberts, 2012), which approximates the complex intractable posterior of the parameter θ given the data X, P(θ | X) with a simple factorized distribution Q(θ), by minimizing the log odds between Q(θ) and the joint distribution P(θ, X) expected under Q(θ), which provides a lower bound for Kullback–Leibler divergence between Q(θ)  and P(θ | X). We performed variational inference for our model using the Python package “pymc3” (Salvatier et al., 2016).

## Results

### Summary statistics

For the control lines across all six accessions, the overall survival rate is 0.42, while the mean ± 1std dry weight in log_10_ mg for the surviving seedlings is 2.86 ± 0.45. Survival rate was determined by whether the plant was present and alive at the time of harvest.

The overall survival rate calculated across the two accessions that received the EMS mutagenesis treatment (Columbia and CV) is 0.45, while the mean ± 1std dry weight in log_10_ mg for the surviving seedlings is 2.60 ± 0.52.

**Figure 2.**
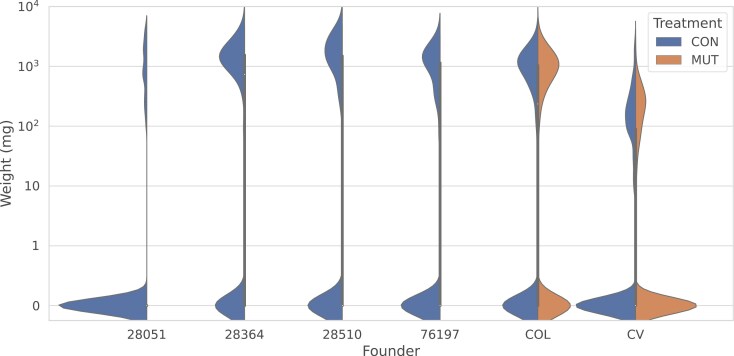
Distribution of dry weight of seedlings for the six founders. For the two founders (Columbia and Cape Verde) that received mutation accumulation treatment, the distributions are separately plotted for the control and the mutation lines. Note that the distributions are bimodal. The probability concentrated at zero corresponds to seedlings that failed to germinate (i.e., dry weight = 0). Density estimation was performed using the kernel density estimation method with kernel bandwidth = 0.09, and grid size = 100. COL = Columbia; CV = Cape Verde; EMS = Ethyl methanesulfonate.

For Columbia (COL), the overall survival rate is 0.544. Specifically, the survival rate for the mutated seedlings was 0.546, compared with the survival rate of 0.508 for the control. The log_10_ mg dry weight of all survived seedlings is 2.909 ± 0.377 for the mutation lines, and 2.891 ± 0.451 for the control lines (see Figure 2; Table 1).

For CV, we observed lower survival rate as well as dry weight than the COL line. Specifically, overall survival rate across both mutation and control treatment is 0.333 (seedling survival rate is 0.332 across all COL mutation lines, and 0.350 for the control line). The log_10_ dry weight of the survived seedlings is 2.222 ± 0.459 for the mutation lines, and 2.143 + 0.531 for the control line (see Figure 2; Table 1).

For CV, 7 out of 17 lines had mean fitness value higher than the unmutated control line. For Columbia, 13 out of 21 lines had mean fitness value higher than the unmutated control line. However, one-tail Dunnett’s test did not detect lines that significantly outperformed the control line for both founders. To account for the zero-inflated distribution in seedling dry weight, we then applied the test to only the survived seedlings for lines with both higher survival rate and mean fitness of the survived seedlings than the control line. Again, no significant difference was detected using Dunnett’s test.

### Posterior estimates of DFE of mutations

Note that since we used a hierarchical Bayesian approach, the parameters of interests, such as the mean and the standard deviation of DFE, have their respective posterior means, variances, and credible intervals. All quantities reported are in units log_10_ mg.

**Figure 3.**
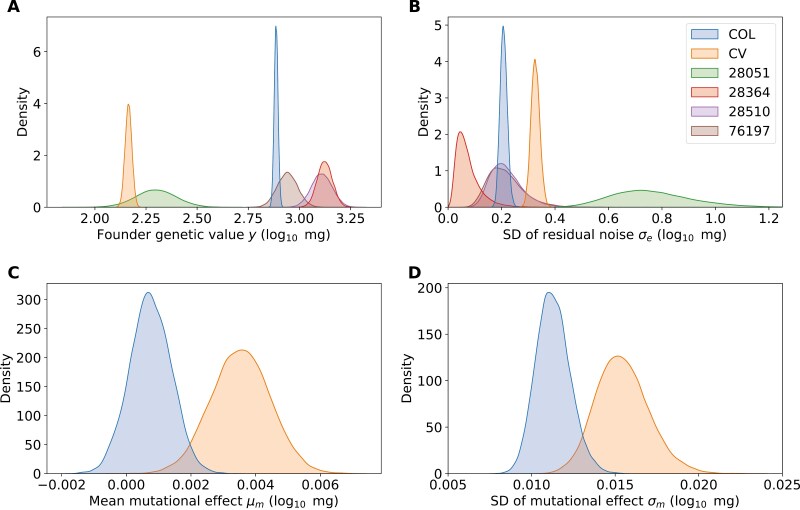
Posterior distributions for various parameters inferred by fitting a Bayesian hierarchical model to the data. (A) founder’s genetic value y; (B) standard deviation of the residual noise η; (C) mean of the distribution of the fitness effect of mutations μ; (D) standard deviation of the distribution of fitness effects, σ. COL = Columbia; CV = Cape Verde. The *y*-axis in all panels is in units of log10 mg.

Overall, our point estimations of the block effects are small, with mean ± 1SD = −0.02 ± 0.02 across all 12 blocks. In Figure 3 and Table 2, we present the posterior distribution of model parameters of the DFE, as well as the residual noise distribution and founder genetic values. The difference in seedling survival rates and the distributions of dry weight between CV and COL can be largely explained by the result that the genetic value (y) of CV founder before the mutation treatment is much lower than COL and other lines. Specifically, the posterior mean ± 1sd of genetic value for CV founder is y=2.164±0.017, compared with y=2.886±0.009 for the COL founder (Figure 3A; Table 2).

**Table 2.** Parameters of the distribution of fitness effects (DFE). The numbers correspond to the posterior means of our Bayesian model. Note that the DFE parameters were only calculated for the two founders, Columbia (COL) and Cape Verde (CV) that received the EMS mutation treatment.

Population	y	μ	σ	η	${h^{2}}_{m}$	*CV*_*m*_
COL	2.886	0.001	0.011	0.208	0.447 × 10^−3^	0.566
CV	2.164	0.004	0.015	0.327	0.337 × 10^−3^	0.775
28051	2.298			0.762		
28364	3.125			0.074		
28510	3.109			0.218		
76197	2.941			0.215		

y : genetic value of the founder. μ: Mean of the DFE. σ: standard deviation of the DFE.

η : standard deviation of the residual noise. ${h^{2}}_{m}$: mutational heritability. ${h^{2}}_{m}=0.16\;\times^{\sigma^{2}}/{}_{\eta^{2}}$. μ, σ, and η are in units log10 mg. COL = Columbia; CV = Cape Verde.

The results for the four founders without mutation treatment are y=2.298±0.099 for accession 28051, y=3.125±0.037 for 28364, y=3.109±0.05 for 28510, and y=2.941±0.05 for 76197 (Figure 3A; Table 2). Note that the posterior distribution for genetic value is much narrower for CV and COL due to their larger sample sizes (control + EMS mutation) compared with the other three founders (*n* = 120 only for the control group).

The posterior distribution of the standard deviation of residual noise (η) is presented in Figure 3B. CV exhibits higher residual noise variance than COL: η=0.327±0.016 for CV; η=0.208±0.013 for COL (Table 2). Here again the posterior distributions of the four lines without mutation treatment are much wider, due to the low sample size.

The posterior distribution of the mean of the DFE (μ) for CV and COL is presented in Figure 3C. The posterior distribution for the mean mutational effect of COL is close to zero, with μ = 0.00075±0.00064, and a 95% credible interval that intersects 0. In contrast, for CV, μ =0.00354±0.00091, with a 99% credible interval not intersecting zero (Table 2). Therefore, mutations in the lower-fitness CV background overall have slightly more positive effects than the higher-performing COL founder.

Finally, in Figure 3D, we examine the posterior distribution of the standard deviation of the DFE (σ) of CV and COL, as a measurement of the average magnitude of mutations. For the Columbia accession, which appears to be more adapted to the experimental field site, σ =0.011±0.001, whereas CV exhibits higher variation in its mutational effects, with σ =0.015±0.002 (Table 2).

We also calculated the per-generation increase in heritability due to mutation (mutational heritability, ${h^{2}}_{m}$) as ${h^{2}}_{m}\;=\;V_{m}/V_{e}$ (Houle et al., 1996), where we first set $V_{e}\;=\;\eta^{2}$. The new variance due to mutation ($V_{m}$) is equal to the variance of the effect of single mutations (σ) times the expected number of mutations per generation in the absence of EMS (diploid genomic rate of mutation rate affecting biomass and fitness = 0.16 per generation, Weng et al., 2019). Using this equation, we found the mutational heritability extrapolated from EMS to new mutations is ${h^{2}}_{m}\;=\;0.447\;\times 10^{-3}$, and ${h^{2}}_{m}\;=\;0.337\;\times \;10^{-3}$, for Col and CV, respectively (Table 2). Assuming that the log_10_ function is well approximated by a linear function close to the founder mean, these values are largely consistent with the previously observed ${h^{2}}_{m}$ for biomass in *A. thaliana* mutation lines (Weng et al., 2021). In addition, we can also calculate the variance in genetic values among the six accessions (namely $V_{g}$) using values in Table 1 and found $V_{g}\;=\;0.174$, which is 8,530 and 4,563 times higher than the $V_{m}$ of COL and CV lines, respectively.

## Discussion

### Distribution of mutational effects

Using chemically induced mutations, we confirm the results of prior *A. thaliana* MA line studies based on natural new mutations that demonstrated the frequency of beneficial mutations is much greater than predicted by prior literature (Lynch & Walsh, 1998). Since our use of EMS precludes the generation of indel mutations, our estimates of mutation effects are restricted to single nucleotide mutations. However, our study, with mutational lines from two founders is consistent with previous *A. thaliana* field studies of 9 founders and their MA lines in a total of 22 founder × field combinations (Roles et al., 2016; Rutter et al., 2010, 2018; Weng et al., 2021) and four greenhouse studies with four different founders (MacKenzie et al., 2005; Rutter et al., 2010; Shaw et al., 2002; Stearns & Fenster, 2016b), i.e., mutations overall are more beneficial than previously expected. Other than Stearns and Fenster (2016b), which reports an EMS approach to inducing mutations, all these previous studies likely estimated fitness across the full spectrum of mutations, i.e., both single nucleotide and indel mutations. Our results are also consistent with several studies that have quantified mutation effects on *A. thaliana* performance in laboratory conditions, where the mutagen is a single T-DNA insert (Murren et al., 2019; Rutter et al., 2017). Spoelhof et al. (2021) found EMS-induced mutations in *A. thaliana* to have uniformly deleterious effects on performance. However, they used a higher dosage and did not transfer the mutations through several generations (as we did).

Transferring mutations across generations eliminates lethal and highly deleterious mutations. A potential limitation of the experimental design is that these mutations are not included in the DFE, but as they are unlikely to contribute to standing genetic variation and therefore may not be relevant to adaptation genetics. A number of other studies with other organisms have documented a higher-than-expected frequency of beneficial mutations since the initial observation that a high proportion of mutations found in *A. thaliana* MA lines may be beneficial (Böndel et al., 2019, 2022; Hall et al., 2008; Latta et al., 2015; Perfeito et al., 2007; Silander et al., 2007), thereby challenging the dogma that mutations are nearly uniformly deleterious (Keightley & Lynch, 2003).

There are at least two explanations for why beneficial mutations are observed with such high frequency in *A. thaliana*. First, if the DFE is highly leptokurtic, as observed by Böndel et al. (2022), then mutations will mostly have very small effect on fitness, but over time a line or lineage will eventually incorporate a highly deleterious mutation that will cause its extinction. Under this scenario, our dosage of EMS, equivalent to 150 generations of mutation accumulation was not high enough to simulate a long enough period to capture those very deleterious mutations. The second explanation is the genotype × environment (G × E) argument that genotypes are rarely at their adaptive optimum, and if mutations have relatively small effect on fitness, then there will be a relatively high frequency of beneficial mutations (Martin & Lenormand, 2015).

Our results are consistent with some theoretical and empirical predictions. Since the four non-Cape Verde and Columbia accessions performed better than either CV or Columbia, we can assume that neither CV nor Columbia accessions were well adapted to our specific field environment. With a uniform fitness surface, we expect a higher proportion of beneficial mutations if the genotype is far from its adaptive optimum (Fisher, 1930), as we have documented here. Mutations in the CV accession exhibit a slightly higher mean effect than those in Columbia, which is also consistent with the adaptive optimum argument since CV has low fitness compared to other accessions in the study area (Rutter & Fenster, 2007). Although we only have two data points demonstrating that beneficial mutations are more likely in the low-fitness founder, this is consistent with previous results with larger number of founder lines (Stearns & Fenster, 2016a). Furthermore, *A. thaliana* populations at the edge of the species distribution harbor more maladaptive loci, likely due to drift and population bottlenecks (Oakley et al., 2023). Since the CV accession is an isolated population, far from the main range of *A. thaliana* in Europe, it is possible that it has a relatively higher genetic load, setting the stage for beneficial compensatory mutations as observed in other mutation studies (e.g., Burch & Chao, 2000).

However, if the DFE of new mutations is leptokurtic (Böndel et al., 2019, 2022), we might have expected to recover severe performance-reducing mutation. But our results do not seem to suggest this. It is possible that our method of mutation induction may have led to a form of somatic selection against deleterious mutations. Cruzan et al. (2022) detected fitness effects of somatic mutations in *Mimulus guttatus*, however, results from *A. thaliana* suggest that somatic selection acting on new mutations is not significant (Monroe et al., 2022). It is important to note that we are unable to reconstruct the shape of the DFE of individual mutations, only the average effect of mutations. This may obscure important details of a stepwise adaptive “walk.” Further, the shape of the mutational DFE has important consequences on evolutionary process from the validity of the molecular clock to how quickly populations can respond to selection via mutations (Böndel et al., 2022). Therefore, future work needs to address both mean effect of new mutations that can contribute to selection response, as well as the exact shape of the DFE.

### Mutational input to genetic variation

While it is clear that standing genetic variation contributes to selection response, it is difficult to quantify the relative contributions of new mutations to this dynamic (Barrett & Schluter, 2008). Our results, based on the production of new mutations that were tested in seminatural environments demonstrate that new mutations are potentially beneficial often enough and of large enough effect size, such that when combined with high mutation rate, they may contribute significantly to rapid local adaptation under natural conditions.

The mean mutational heritability for Columbia and CV is 0.392 × 10^-3^, suggesting that mutation alone could generate typical *h*^2^ of 0.5 (Mousseau & Roff, 1987; Roff, 2012) in about 1,300 generations. Thus, populations can respond to selection pressures via new mutations in a relatively limited number of generations, consistent with previous laboratory investigations of mutational input to *D. melanogaster* selection response (Mackay et al., 1992, 1994).

The variance in genetic value among the six accessions (*V*_*g*_ = 0.174) is 8,530 and 4,563 times higher than the genetic variance generated by mutations alone (*V*_*m*_, see Table 2) of COL and CV lines, suggesting that it would take roughly 4,500–8,500 generations of mutation accumulation alone to produce the observed phenotypic variance among the six accessions at our study site due to genetic contributions (Mousseau & Roff, 1987; Roff, 2012). Furthermore, our results also allow us to estimate the time scale on which CV is able invade the fitness space of Columbia. Specifically, assuming zero mean and constant variance in the DFE over time, the number of generations for the distribution of fitness of CV to contain the mean genetic value of Columbia within 2 SDs from its own genetic value is (*y*_*COL*_ − *y*_*CV*)_^2^/(4 × *V*_*M*, Cape Verde_) = 3417 generations, using values given in Table 2. Thus, low fitness CV ecotype is able to occupy the “adaptive space” of a higher fitness genotype in ~3,400 generations to become equivalently adapted to the habitat, which is on the same order of magnitude as the number of generations needed for establishing the genetic variance among all ecotypes as mentioned above. Overall, we conclude that our results contribute to an increasing number of studies (e.g., Izutsu & Lenski, 2022) demonstrating that new mutations can play a significant role in the evolutionary process at the population level.

We were also able to identify the contribution of “phenotypic noise” (phenotypic variation that is not due to genetic variation). We find that there is greater phenotypic noise in the lower-fitness ecotypes than the higher ecotypes. This fits predictions from recent theoretical work (Rocabert et al., 2020) and corroborates recent experimental work finding that noisy phenotypes can be more beneficial under stressful conditions (Samhita et al., 2021). Although this variation is not heritable and does not contribute directly to adaptation, it may play a role in preventing population extinction during the “waiting time” for additional adaptive mutations (the “look-ahead” effect of Whitehead et al., 2008). Therefore, although phenotypic noise was not a focus of this study, this intriguing result deserves further investigation.

### Novelty and limits of the Bayesian analysis

Our experimental design and Bayesian analysis utilize several novel approaches to estimating the DFE in field environments. Most notably, our inference method is nonparametric, in that it does not assume the shape of the DFE and instead directly characterizes the DFE by estimating the mean and variance of mutational effects. This is thanks to the large number of mutations introduced by the EMS treatment, which allowed us to use the Central Limit Theorem to approximate the joint genetic effect of a relatively large, Poisson-distributed number of mutations. Classical approaches of modeling the DFE usually assume that the DFE follows certain distributions, e.g., the Gamma distribution. However, it has been shown that departures of the true DFE from the assumed distribution (especially if the DFE is bimodal or multimodal) can lead to erroneous estimations of the DFE parameters (Kousathanas & Keightley, 2013). Therefore, hypothesis-free, nonparametric models may prove to be more desirable approaches since they are likely less prone to biases caused by model misspecifications.

Furthermore, we took advantage of the flexibility of Bayesian models to account for several complexities in the data that cannot be fully captured by frequentist approaches. These include explicitly modeling the covariance between sublines due to shared segregating mutations, as well as using a logistic function to model the survival of seedlings as a function of the underlying genetic value, which allowed us to jointly model the zero-inflated fitness data in a principled way. The general Bayesian modeling approach used in this paper may be relevant for other studies of DFEs and may allow researchers to design more complex mutagenesis and MA experiments to characterize finer-scale properties of the genetic effects of mutations.

Our nonparametric approach also contains inherent limitations. Most notably, since we only estimate the first two moments of the DFE, we cannot draw conclusions in terms of the frequency of beneficial vs. deleterious mutations. Recent advances in nonparametric Bayesian theory may be applied to future mutation experiments complete with sequencing data to directly estimate the shape of the DFE from fitness data (Chen et al., 2018, 2021, 2024; Wang & Scott, 2019).

Another potential drawback of our inference procedure is that due to the complex data structure, we used the variational inference procedure instead of unbiased sampling methods such as MCMC. The variational inference method approximates the posterior distribution using simple, factorized distributions to allow fast inference (Fox & Roberts, 2012). Therefore, future work on efficient MCMC methods is needed for fitting data generated from complex mutagenesis experiments with minimal bias.

### Summary

Using chemically induced mutagenesis and Bayesian nonparametric modeling, we estimated the DFE of mutations in field environments for two ecotypes that are differentially adapted to the local environment. We found that mutations can be beneficial (either due to rare large effect mutations or common small effect mutations) for both ecotypes and that the ill-adapted ecotype showed higher variance in the effect of mutations as well as a slight bias towards beneficial mutations in its DFE. Our results suggest that beneficial mutations when combined with high mutation rate and potentially high phenotypic noise can contribute to local adaptation on ecological time scales.

## Supplementary material

Supplementary material is available online at *Evolution*.

qpaf029_suppl_Supplementary_Materials

## Data Availability

Data and codes have been archived in Dryad DOI: doi.org/10.5061/dryad.2rbnzs80w
